# Precision Medicine: An Optimal Approach to Patient Care in Renal Cell Carcinoma

**DOI:** 10.3389/fmed.2022.766869

**Published:** 2022-06-14

**Authors:** Revati Sharma, George Kannourakis, Prashanth Prithviraj, Nuzhat Ahmed

**Affiliations:** ^1^Fiona Elsey Cancer Research Institute, Ballarat Central Technology Central Park, Ballarat Central, VIC, Australia; ^2^School of Science, Psychology and Sport, Federation University, Mt Helen, VIC, Australia; ^3^Centre for Reproductive Health, Hudson Institute of Medical Research and Department of Translational Medicine, Monash University, Clayton, VIC, Australia; ^4^Department of Obstetrics and Gynaecology, University of Melbourne, Melbourne, VIC, Australia

**Keywords:** renal cell carcinoma, gut microbiome, artificial intelligence, precision medicine, nanomedicine

## Abstract

Renal cell cancer (RCC) is a heterogeneous tumor that shows both intra- and inter-heterogeneity. Heterogeneity is displayed not only in different patients but also among RCC cells in the same tumor, which makes treatment difficult because of varying degrees of responses generated in RCC heterogeneous tumor cells even with targeted treatment. In that context, precision medicine (PM), in terms of individualized treatment catered for a specific patient or groups of patients, can shift the paradigm of treatment in the clinical management of RCC. Recent progress in the biochemical, molecular, and histological characteristics of RCC has thrown light on many deregulated pathways involved in the pathogenesis of RCC. As PM-based therapies are rapidly evolving and few are already in current clinical practice in oncology, one can expect that PM will expand its way toward the robust treatment of patients with RCC. This article provides a comprehensive background on recent strategies and breakthroughs of PM in oncology and provides an overview of the potential applicability of PM in RCC. The article also highlights the drawbacks of PM and provides a holistic approach that goes beyond the involvement of clinicians and encompasses appropriate legislative and administrative care imparted by the healthcare system and insurance providers. It is anticipated that combined efforts from all sectors involved will make PM accessible to RCC and other patients with cancer, making a tremendous positive leap on individualized treatment strategies. This will subsequently enhance the quality of life of patients.

## Introduction

Renal cell carcinoma (RCC), an inherently heterogeneous group of cancers, is one of the 10 most common cancers worldwide and accounts for 2% of global cancer cases (1, 2). The incidence of RCC is more common in developed countries, with Belarus having the highest incidence in the world, which has doubled in the last 50 years (2). The incidence of RCC varies considerably among various population and geographical regions. The geographic distribution of RCC shows mainly higher age standardized rate of incidences in Eastern Europe and North America followed by Africa (3). RCC is also known to be influenced by genetic factors, for instance, the risk of RCC is elevated by two-fold for people who have first-degree relative with a history of RCC (4). Genome wide association studies of RCC have shown multiple susceptible loci on the chromosomal regions of 2p21, 2q22.3, 8q24.21,11q13.3, 12p11.23 and 12q24.31 predisposing those harboring these SNPs to RCC (5). RCC accounts for more than 100,000 deaths worldwide each year (6). Most RCC arises from accumulated cell mutation, leading to uncontrolled cell growth in the proximal convoluted tubule located in the cortex of the kidney. It is an insidious and highly heterogeneous cancer, which in most cases is dominated by mutation in the VHL gene function (2, 7). However, RCC is not considered a single entity as it comprises multiple subtypes, each having characteristic histopathological features along with unique genetic traits and clinical outcomes.

RCC, which arises from the nephron tubules is a heterogeneous group of neoplasm having diverse histological subtypes with varying clinical courses and responses to therapy. The histopathological classification of RCC recently has gone through some major changes owing to the key advances in morphological, genetical and epidemiological understanding of RCC subtypes. The 2016 World health organization (WHO) classification of renal tumors is based on the combination of these RCC features and consists of 16 different subtypes and 4 provisional or emerging entities of RCC (8). The 2016 WHO classification includes subtypes that have been classified largely based on their (i) cytoplasmic features; clear cell RCC that arises from proximal tubules and chromophobe RCCs which arises from intercalated cells of distal tubules; (ii) architectural features; papillary RCC arising from distal tubular and mucinous tubular epithelium; (iii) spindle cell carcinoma arising from the cells of the loop of Henle or the collecting duct epithelium; and (iv) anatomical position of the tumors; collecting duct carcinoma arising from the collecting ducts of the kidney and renal medullary carcinoma that develop in the medullar region of the kidneys. The most common subtypes are clear cell RCC (ccRCC), accounting for 70–75% of the RCC cases followed by papillary RCC (pRCC), which is found in 10–15% of cases and chromophobe RCC accounting for 5% of the RCC cases. Oncocytomas comprising of benign tumors accounts for 4–7% of all kidney tumors. Around 4% of the RCCs are unclassified based on the currently available histopathological or molecular parameters. Amongst the new 7 subtypes that were added were based on molecular alterations such as MiT family translocation renal carcinomas, TRCC (originating from the translocation of transcription factor genes *TFE3* and *TFEB*) and succinate dehydrogenase or SDH deficient RCC caused by a biallelic mutation of one of the four subunits of SDH complex; familial predisposition syndrome associated hereditary leiomyomatosis and RCC syndrome associated specific renal disease acquired cystic disease associated RCC. The others that were included as new RCC entities were tubulocystic RCC; small-intermediate size tubules and cystically dilated larger tubules, multilocular cystic renal neoplasm of low malignant potential composed of cysts without expansive growth and clear cell papillary RCC, which shares morphological similarity with both ccRCC and pRCC. The provisional entities of RCC in the 2016 WHO classification include Oncocytic RCC occurring after neuroblastoma (increased risk of RCC after prior blastoma appearance similar to MiT family TRCC), thyroid-like follicular RCC (morphologically similar to the follicular carcinoma of the thyroid), Anaplastic Lymphoma Kinase (ALK) rearrangements- associated RCC (resembles medullary carcinomas and associated with sickle cell trait) and RCC with angioleiomyomatous stroma (sporadic or associated with tuberous sclerosis) (9). Around 4% of the RCCs are unclassified based on the currently available histopathological or molecular parameters (10).

Historically, RCCs are resistant to traditional cancer treatments like chemotherapy and radiotherapy (11). Treatment of RCC depends on the location of the tumor, stage of the disease, and overall health of the patient. Partial or radical nephrectomy (curative surgery) has been the gold standard for treating localized RCC from stages T1b-T4 (12). Ablation and active monitoring by ultrasonography are other popular choices for management of the localized disease. Despite the curative nature of surgery, approximately 30% of patients with localized ccRCC eventually develop recurrence or metastatic disease (13). More importantly, at the time of diagnosis, approximately 30% of patients present with locally advanced or metastatic disease (14). In the late 1980s, cytokines like interferon α (IFN-α) and interleukin-2 (IL-2) were the mainstays of treatment for metastatic RCCs (mRCCs) (15). However, these treatments were ineffective in terms of overall survival (OS), significantly toxic, and linked with high morbidity in patients (16). The subsequent advent of targeted therapies, mainly tyrosine kinase inhibitors (TKIs) such as sunitinib and pazopanib, has revolutionized the management of inoperable and mRCC. Sunitinib, pazopanib, and cabozantinib are TKIs approved as first-line treatments, while axitinib and sorafenib are second-line treatments available for patients with RCC. However, 30% of patients are innately resistant to these treatments, and 70% of the initial responders acquire resistance in 2 years (17, 18).

In addition, a better understanding of the immune system and a recent finding that tumors can exploit immune checkpoints to favor their survival and growth have led to the development of immunotherapeutic agents (ICIs). Immunotherapies in RCC oriented toward programmed cell death protein (PD-1), programmed cell death protein-ligand (PD-L1), and cytotoxic T lymphocyte antigen-4 (CTLA-4) target tumor and immune cells. Recent data from clinics have shown that a combination of targeted therapeutics and immunotherapy agents has increased the median survival for patients with RCC from 15 to 30 months (19).

Despite the progress made in the treatment of mRCC with TKIs and ICIs, most patients (~85%) do not benefit from the current therapeutics and eventually succumb to the disease affecting the overall survival and quality of life (20). The effectiveness of the treatment depends on several factors related to patients, such as geography, socioeconomic condition, type and stage of cancer, patient's age, immune status, and overall general health. The mechanism of action of the drugs used and its interaction with the patient's immune system also contribute to the effectiveness of the drugs used (21). Hence, a more effective way of treating patients with mRCC relies on the development of specific individualized treatments tailored to the patient's explicit needs. The approach commonly termed precision medicine (PM) is gaining momentum in the current arena of cancer treatment. For example, somatic inactivation of the VHL gene is genetically associated with patients with RCC and is one of the major factors regulating RCC pathophysiology. The major effect of an inactivated VHL gene is overexpression of vascular endothelial growth factor A (VEGFA), a key molecule accountable for pathological and physiological angiogenesis-related progression in patients with RCC (22). Agents approved to treat mRCC like sunitinib, which is a standard of treatment in the clinic, have been proven significantly effective as a first-line therapy in patients with RCC harboring VHL gene mutation, as they target VHL gene-associated hypoxia and related angiogenesis regulated mainly by VEGF and its receptors (23). Sunitinib specifically targets the downstream consequences of genetic mutation and, thereby, maximizes the efficacy and minimizes side effects in patients. Despite that, sunitinib resistance occurs in the majority of patients through complementary angiogenic pathways (24). Hence, developing approaches that will not only harness specific genotypes but also other molecular and lifestyle traits of patients are essential to integrating PM into the standard care of patients. This review describes some of the recent progress made in the field of precision medicine (PM)-based approaches in RCC.

In addition, extensive crosstalk between VHL/HIF and PI3K/AKT pathways results in the aberrant activation of PI3/AKT pathway, which contributes to the pathogenesis of RCC (25). In that context, genetic alteration of the PI3K pathway was identified in RCC using a largescale integrated analysis (26). Loss of VHL activation/function with concomitant upregulation of HIF activation facilitates the expression of several growth factors, including VEGF, epidermal growth factor (EGF) and platelet-derived growth factor (PDGF) which activate the downstream PI3K/AKT pathway through their respective membrane bound growth factor receptor. Consistent with that, constitutive activation of PI3K and its downstream components, have been observed in ccRCC (27, 28). This also aligns with the observed activation of downstream AKT, as specified by high phosphorylation levels of AKT and AKT substrates in ccRCC (27, 28). In addition, high frequencies of gene mutations or deletions of *PBRM1* (36%), *SETD2* (15%), *BAP1* (13%), and *KDM5C* (7%) have also been identified in the ccRCC (29). These genes are essential for chromatin remodeling leading to genomic chaos commonly known as ‘chromosomal instability' (CIN), a hall mark of RCC and other cancers (30, 31). In that context, PI3K inhibitor TGX221 has been shown to inhibit the growth of RCC cell lines containing VHL and SETD2 mutations suggesting that VHL/HIF and PI3K/AKT pathways may have a role in the deregulation of chromatin remodeling and CIN, involved with the pathogenesis of ccRCC (32). Considering that small-molecule inhibitors such as sorafenib, sunitinib, pazopanib, and axitinib that target VEGFR, and a new generation of inhibitors (such as brivanib, cabozantinib, cediranib, dovitinib, foretinib, lenvatinib, linifanib, nintedanib, regorafenib, tivozanib, vandetanib, and aflibercept) that not only target the signaling through activated VEGFR but additional targets such as platelet derived growth receptor (PDGFR), fibroblast growth factor receptor (FGFR), hepatocyte growth factor (HGF), and its receptor cMET (33), which are currently being tested in clinical trials for ccRCC patients, which can be contemplated to target the PI3K/AKT pathway in RCC, with the goal to inhibit RCC progression. Hence, developing approaches that will target multiple genotypes in a specific group of patients is essential to integrate PM into standard care of patients. This review describes some of the recent progress made in the field of precision medicine (PM)-based approaches in RCC.

**Figure 1:**
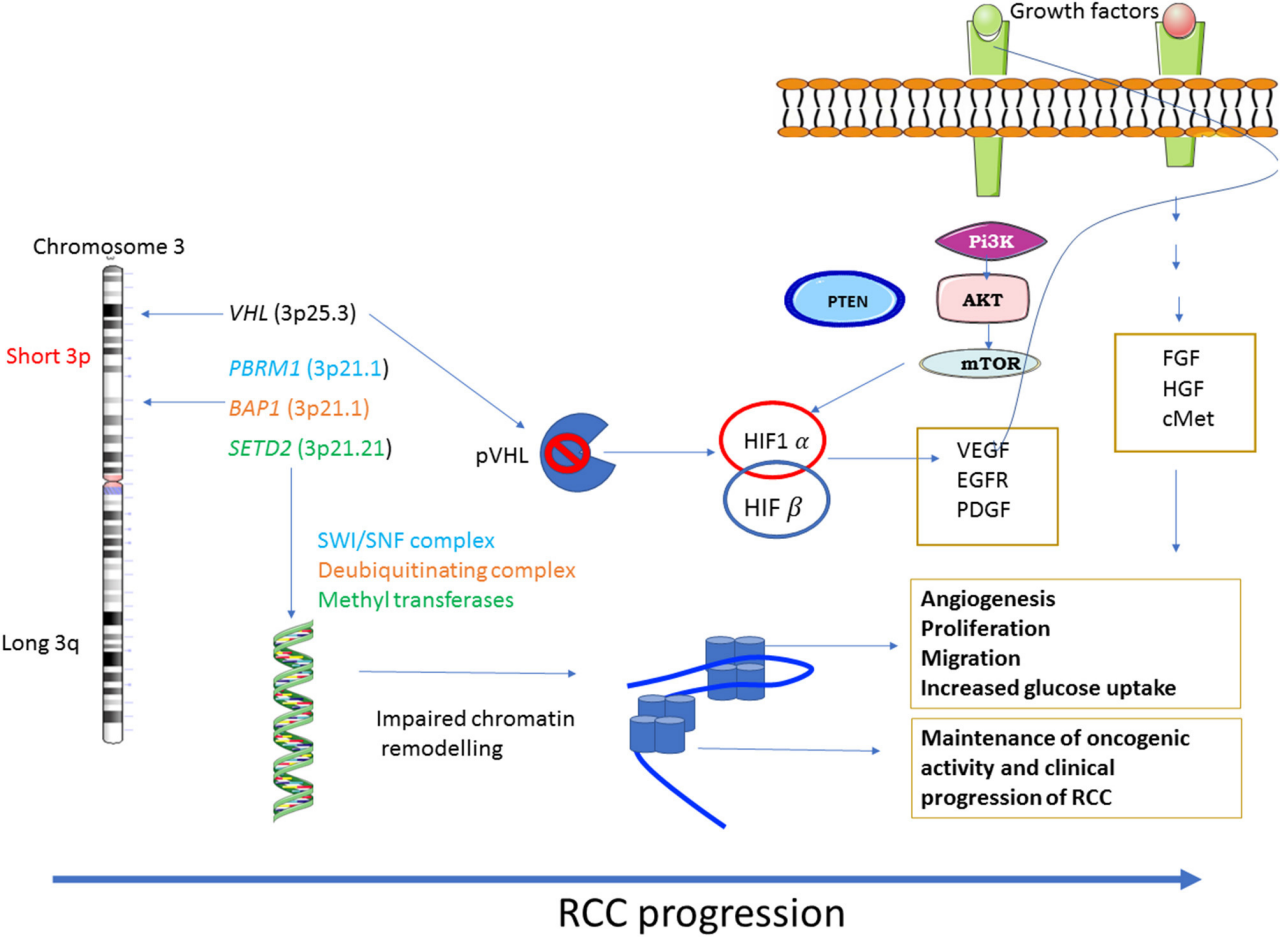
Major dysregulated pathways in RCC: RCC shows a diverse range of genetic mutations. Loss of chromosome 3p tumor suppressor genes play a major role in the pathogenesis of RCC. Genes mostly affected are *VHL*, the gene responsible for sensing oxygen levels within a cell, chromatin remodeling genes such as *PBRM1, BAP1* and *SETD2*. The other signaling pathways that are associated with RCC progression are PI3K-AKT-mTOR and the pathways regulated by FGF, HGF and its receptor c-MET.

Diagrammatic representation of loss of VHL activation/function resulting in constitutive activation of the downstream PI3K/AKT and other pathways is shown in Figure 1.

## What Is Precision Medicine (PM)?

Precision medicine is driven by patient data and refers to the identification of unique patient characteristics, whether genetic, molecular, pathological, or lifestyle, that are recorded and can be selected and used to tailor a targeted treatment protocol for a patient or a group of patients (34). The concept of PM has been used for nearly a century where transfusion patients and their donors were matched for blood or tissue type for transfusion or transplant based on their health records. With the advent of the Human Genome Sequencing Project (HGSP) in 2001, genome sequencing was first made available to clinical practice for the treatment of rare diseases (35). This led to the approval of several gene therapies commonly designed for a group of patients susceptible to a specific genetic disposition. A notable example of this category of patients undergoing treatment currently is the one identified with BRCA mutations for breast and ovarian cancer, human epidermal growth factor receptor 2 (HER2), and progesterone receptor (PR) for breast cancer. These discoveries single-handedly have led to increase in the overall survival and reduced the risk of death by 20% in these patient cohorts (36). In the area of chronic myeloid leukemia (CML), the knowledge that BCR-ABL is the genetic mutation that drives the disease in most patients with CML, which led to the development of a targeted agent that improved survival outcomes in patients (37). In addition, the identification of somatic mutation in the gene encoding the serine–threonine protein kinase B-RAF(B-RAF) in the majority of melanoma cases has provided an opportunity to treat these patients successfully with B-RAF inhibitors (38). Similarly, identification of inherited familial colorectal cancer (CRC) syndromes, such as familial adenomatous polyposis (FAP) and Lynch syndrome [hereditary non-polyposis colorectal cancer (HNPCC)], has led to significant understanding of the molecular pathogenesis underlying sporadic CRC spread and designing of appropriate treatment protocols (39). These discoveries indicate that the PM-oriented development of targeted treatment strategies can realistically benefit many patient groups.

Recent advancements in multi-omics technologies have further accelerated PM-based treatment protocols. Several HGS-like projects are currently in progress across the world by various laboratories and consortiums that may help to strengthen the field of PM (35). A new set of diagnostic assays known as *in vitro* diagnostic companion and/or complementary diagnostics (CDx) is gaining more popularity in recent times. As per the United States FDA, European Regulation (EU) 2017/2016, and Australian Department of Therapeutic Goods Administration (TGA), PM is defined as a test that measures the level of genes, proteins, or mutations that aids in the benefit-risk decision-making about the use of a therapeutic drug, where the difference in benefit-risk is clinically meaningful (40, 41). There are already FDA-approved CDx assays available in the United States, especially for breast cancer, non-small cell lung cancer, melanoma, and CRC. Few examples of CDx assays are the THXID BRAF Kit which qualitatively detects the presence of BRAF mutations in patients with metastatic melanoma by polymerase chain reaction (PCR) (https://www.biomerieux-diagnostics.com/thxidr-braf), Herceptin, HER2 PharmDx Kit based on immunohistochemistry (IHC) and fluorescence *in situ* hybridization (FISH) platforms, which determine the overexpression of the HER2 protein and gene in patients with breast cancer (42).

Over the past few years, the paradigm of mRCC treatment and care has changed drastically. Recently, a lot of effort was spent in integrating molecular targets with histopathology and cancer biology into RCC classification. In 2016, MiT family translocation/Xp11 translocation, fumarate hydratase deficiency, and succinate dehydrogenase deficiency were updated by the WHO after molecular and histopathological reclassification of RCC (9, 43), suggesting that the traditional morphological tumor typing is being replaced by evolving molecular tumor subtyping in RCC. To date, no molecular biomarker with prognostic or predictive value has been approved or is in practice in clinics for RCC; the prognostic stratification of patients with mRCC is still based on clinical factors like low levels of hemoglobin, high serum calcium, increased neutrophil and platelet count, and time from diagnosis to treatment (44). The development of multiple therapeutic approaches focused on different molecular targets in the tumor caters to patients with different subtypes of mRCC and requires the identification of robust predictive biomarkers that will help to classify patients in a way that clinicians can stratify for the right treatment.

## Identification of Predictive Biomarkers in the ERA of PM

The partial success of VEGF-TKI-based targeted therapies in RCC has proved the importance of understanding tumor biology at the molecular level for targeted treatment (45). The introduction of immune checkpoint inhibitor (ICI) has resulted in a paradigm shift in the treatment of RCC patients. In 2015, two different large phase III trials Checkmate 025 (nivolumab vs. everolimus) and Checkmate 214 (ipilimumab plus nivolumab vs. sunitinib) have pushed mRCC into the ICI era (46, 47). Thereafter, a substantial number of trials combining ICIs and angiogenic inhibitors have revolutionized the clinical practice in mRCC (48, 49). As encouraging as it sounds, a significant subset of patients has shown to remain non-responsive (inherent resistance) or stop responding (acquired resistance) to these life-changing treatment options either as single or in combination therapy. As RCC is a heterogeneous cancer both histologically and clinically, where the tumor ranges from a benign to clinically indolent to a most aggressive phenotype with a vast potential to metastasize, identification of predictive and prognostic biomarkers may provide a platform to stratify patients.

### Histopathological Evaluation

Tumor morphology in RCC can be easily underestimated because of the presence of intratumor heterogeneity. This can hinder PM approaches and lead to therapy failure. RCC has been known to show histological heterogeneity across its breadth and the diversity that can be seen in architectural patterns and cytological features. The diversity of clinical behaviors in RCC may occur partly because of pathological and histological variations. A cohort study evaluating the association of ccRCC and nccRCC like papillary and chromophobe RCC with survival showed that, depending on histological subtypes in mRCC, the sites of metastasis differed. The sites of metastasis were also associated with the survival of patients in all histological subtypes (50). Similarly, a significant variation exists within the subtypes of RCC. The phase III trial of sunitinib compared with interferon which pushed sunitinib on to the horizon of systemic therapies available for RCC patients showed that approximately 25% of patients did not respond to sunitinib despite the presence of the clear cell tumor histology. A European clinical trial concluded that the phenotypic heterogeneity seen during the treatment resembles the genotypic and transcriptomic diversity in RCC. Another group identified variation in the treatment responses between subsets of metastases within same patients (51, 52). RCC also exhibits distinct cytological variations, like sarcomatoid and rhabdoid features that are associated with high grade RCCs. Rhabdoid and sarcomatoid features in ccRCC are associated with worse prognosis and poor survival (53, 54). A recent study identified 9 distinct RCC tumor patterns including compact small nests, large nests, bleeding follicles, alveolar, papillary and pseudopapillary, thick trabecular/insular, solid sheet, microcystic, tubular/acinar (55). The RCC subtypes differ in terms of their immune microenvironment for e.g., ccRCC are immune cell rich tumors that respond well to immunotherapy; whereas chRCC and pRCC are immune cold (poor immune cell infiltration), tumors that respond poorly to immunotherapy.

Many studies have shown that the histological pattern in a tumor closely resonates with the molecular features within the tumor (56). For example, ccRCC tumor cells are filled with lipids and glycogen that represents the faulty metabolism associated with fatty acid and glucose breakdown in these tumors. The pathways are altered due to the uncontrolled function of HIF gene that results in the mitochondrial dysfunction that subsequently redirects glucose and glutamine towards glycogen and lipid metabolism (57). For a successful administration of PM to patients an integration of genetical, morphological and molecular data is needed. The role of pathologists has evolved in this era of PM from just identifying and classifying tumors to playing an increasingly involved role in the clinical management of patients. Due to rapid development of technologies and range of different tests undertaken by the pathologists they are able to provide personalized and integrated information to the clinicians who can then provide tailored therapies to their patients (58).

### Histological Biomarkers

Several histological biomarkers (pathological stage, nuclear grade, histology variant, etc.) have been studied across different pathological spectra of RCC. Morphologically, ccRCC is the most common subtype of RCC. However, not all cases of ccRCC have conventional clear cell features with a nest of large uniform cells having clear cytoplasm. High-grade ccRCC tumors do not retain the conventional ccRCC morphology and may contain eosinophilic cytoplasm and papillary or pseudopapillary formation (59). Non-clear cell RCC like papillary RCC contains tumor cells forming finger-like projections called papillae and tubules. Chromophobe RCC consists of cells with atypical nuclei along with granular cells in a solid growth pattern. The remaining uncommon types of RCC show aggressive clinical behavior and a poor prognosis. They are classified according to their unique features; for example, medullary RCC is associated with sickle cell trait, but low-grade oncocytic RCC affects pediatric neuroblastoma survivors (60). In addition, Xp11 translocation renal cell carcinoma, in which the transcription factor gene (TFE3) located on chromosome Xp11.2 is fused by translocation to proline-rich mitotic checkpoint control factor (PRCC) or disheveled segment polarity protein 2 (DVL2), is common in pediatric patients with RCC (61). Most importantly, rhabdoid and sarcomatoid differentiations are features more commonly associated with ccRCC and are associated with worst prognosis (62–64); the higher the clear cell component of a patient's tumor, the greater the chances of the patient benefitting from anti-VEGF therapy (65). Hypoxia-related HIF1α is implicated in the development of RCC (66). In an immunohistochemistry study, complete or partial response to sunitinib was correlated with the expression of HIF1α. Longer progression-free survival (PFS) was associated with lower levels of HIF1α levels (67). Enhanced expression of carbonic anhydrase IX (CA9) and C-X-C chemokine receptor type 4 (CXCR4) has shown promise as biomarkers that can predict the response in patients treated with angiogenic inhibitors. While higher CA9 expression predicted longer PFS in patients with sorafenib treatment, higher CXCR4 predicted poor outcomes in patients treated with sunitinib (68, 69).

### Genomic Biomarkers

The most studied genetic event in RCC is the loss of chromosome 3p that leads to mutations in VHL, polybromo1 (PBRM1), BRCA1-associated protein 1 (BAP1), and set domain containing 2 (SETD2) affecting 90% of ccRCC cases (70). Most biomarker studies have been conducted on VHL, making it the most studied biomarker (71). Although it is the most widely studied biomarker, there is no stable relationship derived between aberrant VHL gene expression and patient outcomes (72). Studies on the predictive and prognostic relationships of VHL mutations in patients treated with anti-VEGF therapy have shown that there is no correlation between abnormal VHL gene expression and the patient's PFS or OS (73). Similar attempts to find a positive correlation between VHL gene expression and patient responses to anti-VEGF treatments showed that, for mutations of VHL, loss of function to be an independent prognostic marker linked to improved response rate, no positive correlation with PFS and OS were reported (74, 75). The presence of PBRM1 or BAF180 tumor suppressor genes encoded at a gene locus near VHL was associated with patients who could be treated for a longer duration with anti-VEGF therapy (76). PBRM1 truncating mutation has been studied much more extensively as a potential biomarker in ICI treatment and has been positively associated with aggressive clinical behavior (77). A pan-cancer study on PBRM1 mutations revealed an association of PBRM1 mutation with OS (HR: 1.24, *p* = 0.47) in 189 patients with mRCC treated with ICI (78). In another independent cohort of the randomized CheckMate 025 trial, the investigators found PBRM1 mutation to be associated with clinical benefits in patients treated with nivolumab (79). Multiple studies found that patients harboring a functional somatic mutation in the BAP1 gene that binds to the BRCA1 and acts as a tumor suppressor gene did not respond well to everolimus and sunitinib treatments as compared to patients with wild-type BAP1 (80, 81). SETD2, which codes for a methyl transferase and is a tumor suppressor protein, has not been associated with significant differences in PFS in patients treated with anti-VEGF therapy (82). However, mutations in the telomerase reverse transcriptase (TERT) promoter region were associated with no clinical benefit in patients (83).

As discussed above, a tremendous amount of effort was spent in identifying putative RCC-specific biomarkers, which can be used as a podium to monitor drug responses. However, there is a serious lack of validated biomarkers for response to mRCC treatments in clinical trials as well as in clinical practice. Proper identification and validation of a robust biomarker panel in addition to the currently used IMDC (International Metastatic RCC Database Consortium) clinical scores are needed to further the goal of PM in RCC (84).

## Integrated OMICS Empowers Precision Healthcare

RCC is a heterogeneous group of diseases, with each subtype having unique and complex biology. To study, analyze, and infer useful insights from tumor biology, it is important to combine and integrate powerful high-throughput tools and available techniques. One such tool is “OMICS,” a multidisciplinary platform that analyzes the interaction and function of biological information obtained from various sets of molecules in an organism such as DNA, protein, lipid, and metabolite. OMICS approaches that integrate and combine data across multiple platforms in a sequential manner to study the interplay of biomolecules in a cancer, in combination with clinical information, can endow clinicians with valuable data to stratify a treatment strategy for an individual patient at a personalized level. This concept is particularly important for non-responsive patients where standard treatments are ineffective, and some extra help in the form of combination therapies are needed to design “patient-specific” protocols that may enable success in better management of those patients.

### Genome Sequencing Technology

The first step in understanding any cancer is to investigate the genetic code and underlying DNA sequence. Hence, as described earlier, differentially and/or unique gene analysis of the patient's samples have been used as an established platform as a genome-based PM in designing suitable therapy for these patients (85). The well-established landmark achievement in genome sequencing technology is the Human Genome Sequencing Project (HGSP). The discovery of 20,500 genes in the normal human genome has paved the way for the development of PM in diseased patients (86). Sanger sequencing and bacterial artificial chromosome techniques were one of the earliest available sequencing techniques (87, 88). However, the more user-friendly next-generation sequencing (NGS) technique that provided a cheaper and effective platform of high-throughput sequencing was later introduced (89). The earliest sequencing data that came out for RCC in 2009 established a higher frequency of mutations in chromatin remodeling genes like lysine demethylase 6A (KDM6A) and SETD2, KDM5C or lysine demethylase 5C and KMT2D or MLL2, and lysine methyltransferase 2D (90). In the following years, PBRM1 and BAP1, genes involved in chromatin remodeling, were discovered to be important drivers of ccRCC by whole genome exome sequencing (WGES) (91, 92). Similarly, in nccRCC, NGS allowed the discovery of unique mutations and somatic copy number alterations (SCNA) in MET proto-oncogene (MET), SETD2, and Neurofibromin 2 (NF2) in pRCC and tumor suppressor genes TP53 and PTEN in chRCC (93, 94).

### Intermediate OMIC Levels: Transcriptomics, Proteomics, and Metabolomics

Although studying the genomics of a disease is an important step in understanding the pathogenesis and progression of the disease, the instruction contained in the gene is transcribed as a functional protein. Gene readouts are commonly known as gene transcripts (messenger RNA/mRNA), and the study of total mRNA is known as transcriptomics. RNA sequencing and microarray are two important tools that allow researchers to know which gene is active and to determine the amount of gene expression in cancer cells. Knowledge of the presence of a gene and its activity aids in understanding the active and inactive pathways in cancer types. Both tools have high-throughput capabilities; however, microarray has proven to be a cost-effective method (95).

Transcriptomic tools have been able to profile RCC subtypes in the past 3 decades. A microarray is a powerful tool that is used to distinguish between clinical subtypes using a very small amount of sample, like in the case of core biopsies (96). Very recently, microarray profiling carried out on liquid biopsies (circulating molecules) is fast becoming an attractive and non-invasive approach for RCC diagnosis and progression of the disease (97). RNA sequencing is conducted to understand signature patterns that provide important data on response to treatments and survival of tumor cells in the heterogeneous RCC tumor microenvironment (TME). Recently, transcriptomic signatures of peritumoral adipose tissues showed that the RCC TME varies depending on the body mass index. This study indicates that the survival impact of obese patients with ccRCC may differ compared to normal-weight patients (98).

Proteomics facilitates the global study of protein profiles across organisms, tissues, or cellular organizations (99). Genomic and transcriptomic profiling provide an understanding of an altered gene sequence or mutation that may be present in tumor cells, but integrating proteomics is crucial as it provides vital information about the functional effect of a particular mutated gene sequence. More importantly, proteins undergo post-translational modification (PTM) after they are translated by ribosomes from the messenger RNA (mRNA) to form mature proteins. However, transcriptomics do not offer comprehensive insights into PTMs that affect the functions and interactions with the cell surrounding (100). The field of proteomics can help in unraveling the alterations in the proteome that are more likely to mirror tumorigenesis and the TME, which is important in PM in order to diagnose and predict diseases. The majority of targets in mRCC therapy are proteins, and conducting protein analysis will result in finding therapeutic targets that have the potential of direct clinical translational capabilities (101).

Two-dimensional electrophoresis was one of the earliest tools used for protein profiling. Multiple studies in the past have leveraged this technique to identify several dysregulated proteins in RCC (102, 103). However, there are several disadvantages of this technique, including the issue of reproducibility. It is also important to mention the low-throughput nature of this technique, which made it less favorable for use as a tool for protein profiling (104). Techniques like flow cytometry or immunohistochemistry have also been used to detect the expression of dysregulated proteins, but over the past two decades, mass spectrometry (MS) has been employed to assess the proteome in RCC (105). The glycolytic enzyme phosphoenolpyruvate carboxykinase 1 (PCK1) and small nuclear ribonucleoprotein polypeptide F (SNRPF) were shown to be significantly dysregulated in ccRCC by liquid chromatography-tandem mass spectrometry (LC-MS/MS). The reliability of LC-MS/MS was validated in ccRCC samples by western blotting (106). The world of proteomics uses two crucial strategies to produce proteomic data, bottom-up proteomics, also called shotgun proteomics (useful for analyzing a mixture of protein) and top-down proteomics (total protein as a start sample) (107, 108). The shotgun method enables to generate a protein fingerprint of individual patients and is an approach suitable for PM, enabling the identification of key biomarkers in patients with RCC (107). A large-scale study identified 596 proteins that were variably expressed in ccRCC in comparison with a normal adjacent kidney tissue. They were also able to validate two proteins, Coronin-1A (CORO1A) and Perilipin (ADFP), that were found to be differentially expressed in ccRCC tissues using immunohistochemistry. Interestingly, while validating, they found that CORO1A was overexpressed in infiltrating lymphocytes and not in tumor cells (109). Middle-down proteomics, which takes advantage of both the shotgun and top-down techniques and uses partial protein digestion to characterize coexisting PTMs, is slowly starting to gain popularity in the field of proteomics. It is emerging as a promising tool for PM as it can explore potential biomarkers by quantifying the expression of a larger number of proteins along with the analysis of individual protein modifications (110). To date, to the best of the authors' knowledge, no study has been conducted on RCC using this new approach.

Studying genetic makeup is an entry point in omics, and the phenotype is the final physical makeup of an organism. Metabolites, along with genetic constitution and cellular microenvironment, constitute the closest reflection of a phenotype of a tumor (111). The metabolome represents all low molecular intermediates or compounds that are products of a metabolic reaction in a cell and, hence, the closest representation of the microenvironment. Studying the metabolome will help in acquiring a better understanding of the cellular process. Metabolomics is a new omics platform when compared to the other omics disciplines (112). It can strengthen PM by being able to predict drug response and safety in a patient (113). Metabolomics is one of the fastest growing platforms and has an upper hand over all the other omics because metabolites are small low molecular-weight substances that can easily be secreted in bio fluids like blood or urine and, hence, can be measured non-invasively (114). Not long ago, nuclear magnetic resonance (NMR) spectroscopy was conducted to analyze metabolites (115). A single RCC study investigated the urinary metabolome profile of patients with ccRCC before and after nephrectomy by NMR. It showed that the levels of creatine, lactate, alanine, and pyruvate were increased, and that the levels of citrate, hippurate, and betaine were decreased in patients with ccRCC in comparison to healthy subjects (116). Another study used the NMR platform and showed that the serum from patients with RCC had elevated levels of very low-density lipids, isoleucine, leucine alanine, N-acetyl glycoproteins, pyruvate, glycerol, and unsaturated lipids along with lower levels of glucose, glutamine, and acetoacetate before nephrectomy and that, interestingly, the pattern was reversed after nephrectomy (117). Recently, a study used a biomarker-based cluster using NMR-based serum metabolomics and self-organizing maps to create an artificial neural network to predict early RCC diagnosis. The study proposed a cluster of 7 metabolites namely alanine, creatinine, choline, isoleucine, leucine, lactate, and valine to validate the metabolomics changes in patients with RCC before and after nephrectomy (118). MS is another data acquisition platform widely utilized to generate metabolomics patterns from biological samples (119). Although an increasingly large number of studies that have used metabolomics to find potential biomarkers are available, there is a high number of studies that have shown an excessive number of false-positive rates as being the greatest challenge in the world of metabolomics (120). However, metabolomics can become a powerful tool in PM when used in conjunction with another available omics platform.

A multi-omics approach integrating various platforms is essential to bridge the wide gap in understanding RCC tumorigenesis that is driven by oncogenes, rewired metabolic pathways, and altered signaling cascades, all leading to a dynamic and, at the same time, defective phenotype. Many studies have highlighted the importance of taking an integrative approach to explore the intertwined association of various biomolecules and its effect on cancer biology, which is the key link in bringing PM approaches to the clinic (121, 122). There are many computational tools now available to integrate different branches of omics, e.g., Metabox (an R-based web application), O-miner, and Galaxy (123–125). From a PM perspective, integrative approaches will help to understand the biology of RCC holistically, thereby improving the ability to predict an early diagnosis as well as drug response in patients.

## Lifestyle-Based Data

Hereditary RCC constitutes approximately 2–5% of all RCCs (126). The remaining RCCs are influenced mainly by lifestyle and environmental factors (127). Cigarette smoking, obesity, and hypertension are established risk factors that are associated with RCC. On the other hand, physical activity and alcohol consumption are known protective factors against RCC (128). Combining lifestyle data with integrated omics data along with clinical and diagnostic information will help scientists generate patterns identifying the risk of developing RCC and predict the disease earlier and will help in determining the most effective intervention against RCC.

## Management and Application of Complex OMICS Data

Acquisition of omics data using high-throughput technologies has allowed the generation of huge amounts of data often in terabytes and petabytes. Notably, The Cancer Genome Atlas (TCGA) contains petabytes of genomics, epigenomics, transcriptomics, and proteomics data (129). To put that into perspective, 1 petabyte is equivalent 223,000 DVDs, each storing 4.7 Gb together. It is estimated that, by 2025, 60 million genomes will be sequenced (130). With huge amounts of data collection come the massive challenge of sorting and storing appropriately. The gigantic amount of data must be stored appropriately so that it is easily accessible by scientists and clinicians for it to be utilized efficiently for clinical diagnosis and treatment. Like the TCGA, there are other publicly available oncology data sets pertaining to patient-based multi-omics data sets (Table 1). The TCGA contains information on 941 renal cancer patient samples (126). The TCGA and other public domain databases aid investigators in combing the vast and diversified omics information into a well-annotated structured data set that may contribute toward stratifying PM for patient groups. There is a range of publications available on RCC that have used these databases to analyze the clinical and biological parameters of patients with the aim of prioritizing structured treatment (131–133).

**Table 1:** Description of multi-omics data repositories related to cancer.

**Data repository**	**Short form**	**Data links**	**Description**
International cancer genomics ***Whole genome analysis***	ICGC	ICGC DATA PORTAL	The repository is a global initiative that provides user- friendly platform for visualizing, querying, and downloading cancer data
The cancer genome atlas ***RNA sequencing, DNA sequencing, single nucleotide variant, copy number variation***	TCGA	TCGA DATA PORTAL	A cancer genomics program spanning 33 cancer types and >20,000 primary cancer and matched normal samples
Clinical proteomic tumor analysis consortium ***Mass spectrometry-based proteomics data***	CPTAC	CPTAC DATA PORTAL	A proteogenomic Cancer Atlas of comprehensive sequence of proteomic datasets
Cancer cell line encyclopedia ***Gene expression, mRNA expression SNP genotyping, pharmacological profiles of 24 anticancer drugs***	CCLE	CCLE DATA PORTAL	The project validates >1000 human cancer cell line models by detailed genetic and pharmacological characterization
Therapeutically applicable research to generate effective treatments ***Gene expressions, copy number, miRNA expressions***	TARGET	TARGET DATA LINK TARGET DATA MATRIX	TARGET applies a comprehensive genomic approach to determine molecular changes that drive childhood cancer
Cancer genome characterization initiative ***Gene expressions, copy number, sequencing data***	CGCI	CGC1DATA PORTAL CGC1DATA MATRIX	CGCI uses molecular characterization to uncover distinct features of rare cancer
Omics discovery index ***Genomics, transcriptomics, proteomics, and metabolomics***	OmicsDI	OMICSDI	The tool provides a framework across heterogenous omics datasets
Molecular taxonomy of breast cancer international consortium ***Single nucleotide polymorphisms, Gene expressions, computational biology***	METABRIC	MOBCCRC	Breast cancer PM and Computational Cancer Biology programs incorporates multidisciplinary techniques to develop statistical models to understand genomic abnormalities

The key challenge in using these multi-omics approaches is to understand, interpret, apply, and translate the knowledge generated from the huge omics data. According to the International Medical Informatics Association (IMIA), bioinformatics techniques and algorithms are used to analyze and extract the hidden knowledge in the diverse and complex “Big Data” (134). Although harnessing Big Data related to patients is essential in making the PM approach in oncology a success, it can come with its share of adversities in patients. Collection of Big Data about patients from different sources and networks may, in certain cases, result in data theft and misuse, leading to uncertainty, social discrimination, and biases for patients, which again can result in undesirable health effects for patients.

## Nanotechnology-Based Approaches in PM for RCC

One of the challenges in cancer therapy and more so in RCC is the broad non-specific target-based treatment approach, which not only causes severe side effects in patients but also in most cases enhances drug resistance by enhancing the survival of chemotherapy-treated residual tumor cells into an aggressive phenotype. The advantage in the application of nanotechnology is the use of “nanocarriers” as drug delivery vehicles that mediate targeted delivery of drugs to tumor sites without causing much harm to normal tissues (135). However, the technology is still in infancy and, currently, there are a few clinical applications of this technology in cancer treatment [127, 130–133]. Nonetheless, several *in vitro* and *in vivo* mouse model studies have demonstrated that the technology has the potential to have a significant impact on clinics.

Nanocarriers, usually having a size of less than 100 nm in one dimension, are organic/inorganic or hybrid particles shaped in the form of micelles, dendrimers, liposomes, or virus-like particles and are used to encapsulate/covalently conjugate or absorb cancer drugs. These nanocarriers have shown significantly greater efficacy in different cancer models compared to drugs on its own (136). Nanoparticle formulated (nanocarriers) drugs allow for specific targeting of tumor cells by providing superior solubility and stability of drugs in the tumor microenvironment, resulting in improved internalization of the drugs in the tumor without much loss in the circulation, circumventing harmful side effects in patients. One great advantage of nanoparticle-formulated drugs is that they cannot pass the tight junctions of the normal vascular lining but can easily pass through the leaky vascular lining of tumors to enhance their concentration at the tumor site (137). This phenomenon known as “enhanced permeability and retention effect (EPR)” is the fundamental principle of nanoparticle-conjugated drug treatment of tumors (138). However, in that scenario, the surface area and size of nanocarriers play an important role in active tumor targeting and are adjusted for EPR effects to occur without unwanted uptake of nanodrugs by the normal endothelial system (138). Common examples of these formulations are Abraxane (albumin conjugated paclitaxel), PEGylated (polyethylene glycol formulated), doxorubicin (DOX), and DOXIL recently approved by FDA for cancer treatments (139, 140). These nanodrugs have shown enhanced efficacy in patients with less cardiotoxicity compared to conventional drugs. In addition, active targeting of tumors by nanoparticle-conjugated drugs also occurs by physically attaching the surface of nanocarriers to certain overexpressed antigens on the surface of tumor cells. Tumor-specific cell surface overexpressed antigens with nanoparticle-formulated antibody conjugation have recently been shown to target folate receptor overexpressing prostate, breast, and lung cancer cells *in vitro* (141). Moreover, the application of nanoparticle-conjugated epidermal growth factor receptor (EGFR) and HER2 receptors on different cancers leading to increased efficacy of different cancer drugs have recently been demonstrated (142, 143). Besides these, the cluster of differentiation (CD), estrogen, integrin, and other growth factor receptor-based targeting using different nanoparticles have successfully been shown to enhance the efficacy of current cancer drugs *in vitro* and *in vivo* mouse models (144–146). In cases where there is poor penetration of antibodies inside cells, antigen fragments (Fab) or single fragments (scFv) are also used to overcome these deficiencies.

As such, nanomedicine has the potential to empower PM in oncology, as the key step is to select the right drug delivery platform for a patient cohort that can target specifically overexpressed tumor-specific protein sets aberrantly regulated by cancer. Human kidneys are an ultrafiltration unit and are responsible for filtering the circulating blood. In that case, the shape, size, and charge of nanoparticles are important factors when designing nanocarriers for RCC. In that scenario, cationic spherical nanoparticles with diameters between 6 and 8 nm have been shown to have better renal clearance (147, 148). Pre-clinical studies focusing on RCC have concentrated on selectively targeting kidney tumors (149, 150). Tumor hypoxia is a leading cause of drug resistance in RCC (151). Recently, it has been shown that certain nanoparticles can be activated under hypoxia-induced oxygen stress. Among those, hypoxia receptive electron acceptor nitroimidazole conjugated with carboxymethyl dextran and loaded with DOX showed accelerated release of DOX to hypoxic tissues *via* the EPR effect (151, 152). In addition, iron oxide conjugated DOX linked with azobenzene-4, 4-dicarboxylic acid released 80% of DOX in hypoxia compared to only 10% release in normoxic tissues (153). There are nanocarriers that have demonstrated the application for targeting hypoxia in tumors, which may have clinical application for RCC (154–156). In addition, few gold, silver, silica, and iron-based nanoparticles including liposomes have shown applicability in targeting angiogenesis, which is a major decisive factor in RCC progression and resistance (157). In that context, tumor necrosis factor (TNF)-mediated secretion of vascular endothelial growth factor (VEGF) was decreased by curcumin-loaded PLGA nanoparticles (158). In a hepatocellular cancer model, chitosan nanoparticles suppressed the expression of VEGFR2 with subsequent suppression of VEGF synthesis, leading to an anti-tumor effect in the mouse model (159). These and other nanoparticle-based studies targeting hypoxia and angiogenesis hold a great promise in increasing the efficacy of current drugs used for RCC treatment. However, significant challenges exist in modulating these anti-hypoxia and anti-angiogenesis nanomedicine-based approaches successfully *in vitro* and in mouse model studies with consequent application to patients with RCC.

Recently, nanoparticles have been used in RCC with an intention to reverse sunitinib resistance. Cuprous oxide nanoparticles (CONPs) can downregulate the expression of AXL, MET, AKT, and ERK to improve responsiveness to sunitinib in resistant RCC cells and may be a less toxic way to treat patients with acquired sunitinib resistance (160). Although few nanomedicines such as liposome-based Onivyde and Vyxeos have been approved to treat certain solid cancers (pancreatic, esophageal, and colorectal) (161), there are no FDA-approved nanoparticle-based drugs for RCC. Zinostatin stimalamer, a lipophilic analog of the antitumor antibiotic zinostatin, is the only approved nanomedicine-based drug available in Japan for RCC treatment (162, 163). CRLX101, a novel nanoparticle-drug conjugate containing camptothecin and inhibitor of topoisomerase I, HIF1, and HIF 2α was tested recently in a randomized phase II trial along with the anti-angiogenic drug bevacizumab vs. the standard of care for mRCC like bevacizumab, axitinib, everolimus, pazopanib, sorafenib, and sunitinib. Unfortunately, the combination failed to demonstrate any improvements in the PFS of patients with mRCC when compared to standard treatments (164).

## Artificial Intelligence in PM

Artificial intelligence (AI) is an arm of computer science that makes use of computer-generated data to mimic human intelligence. RCC is a multi-faceted disease with many genetic and epigenetic variations, and AI algorithms can push forward personalized RCC detection and diagnosis in leaps and bounds toward the autonomous disease diagnosis field with the help of big data sets. Machine learning (ML) and deep learning (DL) are driving today's AI advancements. ML is an important type of AI that can learn from a whole heap of data and make predictions. DL is a subset of ML that uses an artificial neural network that mimics the human brain's information processing approach. A well-planned DL can diagnose and classify diseases and make predictions with high accuracy.

AI together with ML and DL can improve RCC diagnosis and treatment in digital healthcare. Most patients with RCC are diagnosed incidentally when scanning for other diseases. However, there is no sure way of predicting that renal masses are cancer by imaging alone. Tissue biopsy is a gold standard in many cancers; however, renal mass biopsy has significantly higher non-diagnostic rates and fails to aid in diagnosis (165, 166). Approximately 20% of small renal masses (<4 cm) are non-malignant and do not need surgeries but still end up undergoing partial or full nephrectomy (167). Differentiation between small renal masses and RCC is an important aspect of patient management, and this is where AI can play an important part in PM. AI algorithms can be used to accurately predict whether the renal mass shown in patient scans is cancer or not (168, 169). Many research studies have developed complex neural networking programs that can process digitized renal histopathology slides and learn patterns to identify tumors (170). AI has touched every aspect of the prognosis of patients with RCC right from diagnosing RCC to predicting prognosis and recurrence in patients (171–173). To accurately tailor-make treatments for patients with RCC, an important step is to accurately predict drug response in individual patients. Therapeutic resistance, both innate and acquired, is a financial burden in any disease. Thirty percent of patients with RCC are known to be innately resistant to the targeted therapy, and another 30% respond initially, develop resistance later, and show up with increased tumor burden (174). RCC researchers have very recently designed deep neural networks to predict tumor drug response (175). ML algorithms have perfectly predicted the chemoresistance of cancer cell lines (176). AI is already gaining momentum in clinical medicine, specifically in medical imaging. In early 2021, the FDA released the agency's first action plan named the “AI/ML-based software,” which outlined the FDA's next steps toward the oversight of AI/ML-based medical action (177). Very recently, the FDA approved GI Genius, the first device that uses AI/ML to assist clinicians in real-time detection of polyps or tumors in the colon (178). Few AI algorithms like WRDensity, HealthMammo, Profound AI, and Transpara have already been approved in breast cancer that help radiologists and clinicians in identifying suspicious mammograms (179, 180).

The advantage of AI/ML-based programs is that they are continuously evolving with new data sets. Harnessing its true potential is important to achieve the goal of PM where it can help healthcare systems to automate tasks that are time-consuming and overwhelmingly difficult for physicians.

## The Role of the Gut Microbiome in RCC PM

Over the past decade, considerable advances have occurred in understanding the implication of the gut microbiome in normal biology and how it changes with tumor initiation and progression through manipulation of the immune system (181). In the human body, extensive interaction exists between host cells and millions of symbiotically blossoming microbes. These symbiotic microbes colonize in different organs of human bodies and undergo constant changes triggered by endogenous and exogenous stimuli. However, substantial portions of these “healthy microbes” remain unidentified by current microbiological techniques. Nonetheless, distinct “dysbiotic” microbiome signatures have been associated with cancer and other diseases (182). Changes in the dysbiotic gut microbiome occur with initiation of cancer in patients in response to surgery and chemotherapy treatments and are associated with cancer recurrence and contribute substantially to the efficacy of cancer therapies (183, 184). A plethora of integrated omics studies have emphasized the role of gut commensals in tumorigenesis and cancer treatment (185–187). Recent studies also suggest that host-microbiome signature can even correlate with survival parameters in patients with cancer, suggesting that the modulation of gut microbiome signature can potentially dictate the success of cancer therapy (188, 189). Hence, the gut microbiome signature is rapidly becoming a target for new treatments and diagnostic models.

In RCC, gut microflora are definitely an important non-genetic contributing factor to the progression of the disease, as microbial populations in the gut of patients with RCC have been found to be distinct from their adjacent normal tissues (190, 191). Gut microbiota can also modulate the tumor microenvironment (TME) by influencing the levels of various metabolites such as dietary amino acids and short-chain fatty acids, for example, butyrate, acetate, and propionate, which have an anti-inflammatory property (192). Immunotherapy treatment founded on ICI-based PD-L1 and PD-1 antibodies has changed the landscape of RCC treatment (193). However, primary resistance occurs in the majority of patients with RCC. Recently, studies on mice xenografts and on patients with cancer have shown that abnormal gut microbiome profiles can influence primary resistance to ICI treatment (194). Particularly, an antibiotic therapy prior to ICI treatment in patients with RCC significantly reduced the overall and progression-free survival compared to a cohort of patients who were not treated with the antibiotic (195). In univariate and multivariate analyses, antibiotic treatment was a single prognosticator of ICI treatment failure in these patients. Using quantitative metagenomics, the composition of the gut microbiome of patients participating in the study was analyzed. Positive clinical outcome in responding patients with RCC was associated with the enrichment of the *Akkermansia muciniphila* (*A. muciniphila*) microbe in the gut microbiome (177). Further studies are in progress in which fecal bacteria from patients responding to ICI treatment will be transferred to non-responding patients. Such treatments have been performed with success in other groups of patients with cancer (196). In addition, the microbiota of non-responding patients can be controlled by diet and use of prebiotics for a favorable outcome in patients in response to therapies. Hypothetically, if the prevalence of microbial population in non-responders can be changed toward the microbiome profile in responders, that would enable the non-responders to respond to ICI therapy (197).

In the same context, a shotgun DNA sequencing technique was used to screen the gut microbial composition of patients with advanced RCC (198). It was found that TKIs given to patients prior to ICI treatment shifted the gut microbiome profile of patients positively and enhanced the growth of immunostimulatory microbes like *Alistipes senegalensis* and *Akkermansia muciniphila*. Hence, harnessing the positive effect of gut microbiota in patients with RCC can enhance the clinical efficacy of ICI therapy (198). Few other studies have also emphasized the importance of modulating gut microflora to achieve successful outcomes while using ICIs on patients with mRCC (199, 200).

With respect to PM, microbiome-based treatment approaches are gaining momentum where individual host-microbiome patterns can be integrated with other personal health-related information of a patient for evaluation if the microbiome-based PM approach will be a suitable treatment option. As analyzing and storing of individual patient's microbiome profile will be challenging, AI in that scenario may play an important role in finding essential clinical evaluations, thereby promoting the field of PM to evolve. In that context, the application of AI in predicting chemotherapy resistance in patients with ovarian cancer based on gut microbiota profiles has recently been reported (201).

Although evaluation of the microbiome-cancer axis has started, a lot of heavy lifting is needed for translational possibilities. There is an extensive diversity of gut microbiomes in individuals depending on their genetic makeup, diet, and diverse lifestyle. Adding to that complexity, microbes interact with the host and the tumor in a diverse and context-specific manner. Hence, elucidation of microbiome composition and unique mechanisms by which the gut microbiome interact with the host and tumors may prove challenging for PM. However, a significant breakthrough in microbiome-based diagnostics and therapeutics is underway based on the identification of unique tumor-specific gut microbiome and/or metabolic signatures combined with existing immune cell types. This could potentially stratify patients into different risk groups and may facilitate the prediction of models for early-stage screening (202–204). In these scenarios, big data analysis using AI/ML algorithms may play significant roles in developing precise and reliable prediction, diagnostic, and therapeutic tools for cancer treatment.

## Cancer Vaccines and Cellular Therapies: Promising PM Strategies in RCC

Vaccines have been, for a very long time, thought to be a viable treatment for cancer. The first published literature on oncology vaccines dates to 1893 when an inoperable soft tissue sarcoma was targeted by injecting streptococcal toxins that enhanced a non-specific immune response in a patient (205). Many cancer vaccines are in clinical trials with the aim to enhance patient's immune response against tumor cells. Vaccine therapy can be implemented if a tumor is immunogenic, and RCC is a proven immunogenic tumor, as there is significant infiltration of lymphocytes in the tumors (206). A recent phase 1 clinical trial studied the safety and efficacy of autologous dendritic cells transduced with AdGMCA9 (GM-granulocyte macrophage colony-stimulating factor + CAIX delivered by an adenoviral vector) and was found to be well tolerated without any significant safety concerns in patients with mRCC (207). Neoantigens or mutation-generated novel epitopes of self-antigens in tumor cells have recently been proven to have greater potential than tumor-associated antigens like CAIX. PM tools like NGS have made it possible to identify several neoepitopes in individual patient tumors that are potential targets to develop treatments. GEN-009 is a personalized neoantigen vaccine that is in the phase 1/2a trial (NCT0363310) for RCC and other solid tumors. Patients who received PD-1 based immunotherapies were selected for the NCT0363310 vaccine trial. GEN-009 was able to enhance T cell responses in 100% of evaluated patients. The trial emphasized that the approach of combining vaccines with ICI has a good therapeutic potential. GEN-009 consists of neoantigens that were identified based on a cell-based bioassay platform that uses patient's own monocyte-derived dendritic cells to identify neoepitopes (208). Although GEN-009 is still far from clinical use, the excellent results of phase 1/2a is a perfect example that vaccine-based PM strategies can be tailored toward specific patient groups or individuals.

Chimer antigen receptor (CAR)-T cells are genetically engineered autologous T cells to express CAR that can specifically recognize tumor cells. CAR-T cells, after their success with hematologic malignancies, have been proven to be an illustrative example highlighting the use of the PM platform to improve patient outcome. CAR-T cells are tailor-made for patients using their own white blood cells and, so far, are approved for treating leukemia and lymphoma. KymriahTM (relapsed or refractory acute lymphoblastic leukemia and large B-cell lymphoma), YescartaTM (lymphoma), and TecartusTM (relapsed or refractory mantle cell lymphoma) are the only three autologous CAR-T cell treatments that are currently approved by the FDA and have reached the market (209, 210). CAR-T cell therapy in the case of hematologic cancers has been particularly successful because it harnesses CD19, a unique protein only found in hematological malignancies and is not expressed by solid tumors. As RCC tumors express proteins that are also expressed by healthy kidneys, the potential application of CART-cell therapy may not be an option for patients with RCC until RCC-specific proteins are discovered that can be targeted by CART-cells.

Recently, human endogenous retrovirus E (HERV-E) derived antigen was found to be expressed in majority of ccRCC cells with no expression in normal healthy tissues. A phase I clinical trial based on this finding is currently actively recruiting patients to study the safety and efficacy of HLA-A11:01 restricted HERV-E specific CART-cells in patients with mRCC (NCT03354390). Another clinical trial currently recruiting patients is a phase 1/1b open-label multi-center study that will analyze the safety and efficacy of TRQ-1501 in patients with relapsed or refractory solid tumors like RCC (NCT03815682). TRQ-1501 is an immunotherapy developed from patient's own T-cells capable of targeting heterogeneous tumor antigens in addition to overcoming immunosuppression in TME. TRQ-1501 is also loaded with 1L-15, 1L-12, and TLR agonists whose prime function will be to activate the immune system. Another clinical trial that is worth mentioning is NCT03393936, an umbrella trial that is a phase 1 and 2 trial studying the safety and efficacy of two CART-cells, CCT301-38 and CCT301-59, in relapsed and refractory patients with stage IV mRCC. CCT301-38 will target AXL on tumor cells while CCT301-59 will target receptor tyrosine kinase-like orphan receptor 2 (ROR2) antigens in tumors. Patients in these trials are selected based on the expression of tumor antigens they express. Although all the above trials are in their infantry, they sure are a step ahead toward the PM era in RCC.

## Circulating Tumor Cells and Patient-Derived Tumor Organoids: Their Relevance to PM in RCC

The trans-circulatory pathway is one of the most important pathways in RCC metastasis (211). A metastatic disease is the result of circulating tumor cells (CTCs), a rare subset of tumor-disseminated cells that are shed in the patient's blood. CTCs maintain the inherent primary tumor properties and can be detected very early as cancer progresses, making them a powerful clinical biomarker. RCC, being a highly invasive cancer, can benefit from the early detection of RCC-specific CTCs. The CellSearch circulating tumor cell test is the only FDA-approved CTC detection test used in clinics. Baseline CTC detection has been proven to be an important prognostic marker of PFS and is a significant predictor of poor response to tyrosine kinase inhibitors in patients with mRCC (212). CTCs can be a good candidate for a preclinical model because they retain the heterogeneity and properties of primary tumors. These models can then be tested for drug screening and disease modeling (213).

The potential PM application for RCC drug and biomarker screening could be generating patient-specific pre-clinical 3D-organoid models. Patient-derived 3D organoids have been developed from primary tumors and CTCs in various cancer types (214, 215). Primary tumor and CTC-derived explants have recently been studied in *in vivo* drug-resistant models. Analysis of the genetic makeup from resistant models and relapsed patients can provide a clearer picture about the development of resistance in cancer (216). A clinical study on treatment guided by patient-derived xenografts (PDXs) could identify an effective treatment regimen for 11 out of 12 patients with advanced cancer types (1 patient died before receiving treatment) (217). Thus, 3D organoids and PDXs have immense potential for a guided treatment regimen. Although the studies require further development, CTC, patient-derived 3D organoids and PDX are in use for the development of PM-based cancer therapies.

## Current PM-Based Target Therapies and Immune Checkpoint Inhibitor Strategies in RCC

Renal cell carcinoma, in terms of available treatment, has come a long way from being a therapeutic orphan to one with multiple treatment options. RCCs are innately resistant to chemotherapy and radiotherapy. The exponential growth of research on RCC has led to the clinical knowledge that RCC has mutated pathways like the VHL pathway that sustains RCC cell growth by supporting angiogenesis, and the PI3K-Akt-mTOR pathway that supports the progression of the disease (24). Not surprisingly, targeting angiogenesis as well as the mTOR pathway with angiogenic inhibitors and mTOR inhibitors have shown tremendous improvement in the overall response of patients with RCC. However, as mentioned before, only selected patients can reap the benefits of these treatments. Another PM-based approach in RCC has been the use of monoclonal antibodies that block the immune checkpoints. RCC is an immunogenic tumor; tumor cells evade the immune system by overexpressing the immune regulators that keep autoimmunity and self-tolerance in check (218). PD-1 and PD-L1 are transmembrane immune and tumor cell regulatory proteins that modulate activation or inhibition of immune cells (219). PD-L1 tumor expression is a poor prognostic factor but a good response predictor for the use of both PD-1 and PD-L1 inhibitors in RCC (220). A recent meta-analysis study on the expression of PD-L1 has shown that patients with RCC harboring high expression of PD-L1 in tumors responded significantly better to both PD-1 and PD-L1 antibody therapies compared to patients with low or negative PD-L1 expression in tumor cells (221). Each PD-1/PD-L1 drug approved by the FDA takes into consideration PD-L1 expression based on an immunohistochemistry (IHC)-based tissue assay. Only a small fraction of patients with a negative PD-L1 expression by IHC assay showed any response to PD-1/PD-L1 antibody therapy. This suggests that the identification and utilization of PD-L1 expression in tumors is of great significance for a better selection of patients who may respond to PD-1/PD-L1 therapy.

More than dozens of immune checkpoints have been identified over the past decade, nivolumab, anti PD-1, was the first immune checkpoint inhibitor (ICI) that was approved by the FDA as a monotherapy for treating advanced RCC in 2015. Thereafter, in April 2018, a CTLA-4 inhibitor, ipilimumab, in combination with nivolumab, was approved by the FDA for intermediate and poor risk in previously untreated patients. Soon, anti-VEGF/ICI combination treatments appeared to produce favorable outcomes in patients with RCC. As a result, in 2019, two combinations, pembrolizumab (anti-PD-1) plus axitinib (tyrosine kinase inhibitor) and avelumab (anti-PD-L1) plus axitinib, made their way as FDA-approved drugs in mRCC treatment plans (193). As with any other treatment, the success of ICI is also very vague, so effective use of ICIs with respect to PM needs robust predictive biomarkers that can predict response to tumors. Biomarkers that have claimed to predict ICI responses include PD-L1 expression, tumor mutational/neoantigen burden, microenvironment signatures, and occurrence of immune-related adverse events (222). Recently, a study showed that the frequency of PD-1^+^CD8^+^ T cells relative to PD-1^+^ regulatory T cells or Tregs in the TME is a far superior biomarker that can predict the efficacy of anti-PD-1 treatments than PD-L1 expression and tumor mutation burden (223). Other studies that are gaining increased momentum in recent years have highlighted the success of re-challenging patients treated with ICI to explore its safety and efficacy (224). If ICI re-challenging studies translate to clinical settings, research on exploring the identification of potential biomarkers will be in high demand for the selection of the right group of patients.

## Regulations for the Implementation of PM

PM is an opportunity to treat individual patients with cancer at a greater targeted therapeutic resolution. With the rapid shift of cancer therapeutics toward the use of PM in patient care, there is a greater need of refined regulatory guidelines to ensure the best and safe PM-based patient care. Many countries like Australia have very recently introduced regulatory laws revolving around the use of PM, while countries like the United States and European countries modified their regulatory landscape to include regulatory laws pertaining to PM. Most regulatory approvals are recognized by the FDA and Centers for Medicare and Medicaid Services (CMS) in the United States and the European Medicines Agency in the European Union (225). Different centers under the FDA and CMS regulate the approval of PM as per their jurisdiction and regulations. For example, laboratory diagnostic tests are regulated by CMS. Similarly, in the European Union, the EU regulatory framework for pharmaceuticals offers several different legislations that regulate the development of PM. *In vitro* diagnostics and medical device legislations aim at adapting the EU legislation to the technological and scientific progress in the PM sector and provide a better consultation process for companion diagnostics, similar to clinical trial regulations that aim to simplify the conduct of clinical trials and research in therapies using PM (226, 227).

The regulatory scenario for PM is changing worldwide to accommodate its fast-changing landscape. In 2018, 42% of all new drug approvals by the FDA were personalized therapies (228). However, PM has multiple moving parts, and that not only involves PM-based drugs but different services, devices, and associated technologies that currently are regulated by multiple agencies, with no clear regulatory framework associated with PM treatment in patients. With the PM era pacing rapidly in the direction of modern medicine, there is an urgent need for associated regulatory boards to continually update and adapt to maintain the high quality and safety of PM-based treatments and associated products and technologies.

## The Downside of PM

PM is a young and rapidly growing field and, like any new scientific/therapeutic field, personnel involved are in uncharted territories. Although PM has the potential of touching a patient's life and be able to make a positive change to their health, it still must make a huge amount of progress in order to fill the gaps between the research, clinical trials, and clinics for successful therapeutic application in patients.

### Privacy Concerns and Ethical Issues

One of the foremost issues is the amount of patient data required, generated, and stored for the proper implementation of PM. This can lead to privacy issues related to handling and storing the massive data sets. The use of technologically big data-generating tools like the NGS and OMICS platforms may worsen this issue further. To apply PM successfully, integration of NGS- or OMICS-generated data along with patient's other health records including diagnosis, laboratory works, and demographic information is needed. Patient data may differ depending on how they are handled, collected, and stored (229). Generation of error-free clinical data and their recording and interpretation are of prime importance. Digital health records and networks are used increasingly to ease the daunting task of storage of large volumes of patient information (230). Generation of large amounts of data like whole-genome sequencing (WES) of a patient may raise concerns related to privacy acts and discrimination. In addition, the big data sets generated by the NGS, WES, and OMICS platforms need treating physicians to have special interpretation skills. In that context, treating physicians will need adequate genomic knowledge and training for data interpretation, as these data sets will formulate prescribed treatments for patients. The issue may be exacerbated further by genomic findings related to “variants of unknown significance” or “false positives/negatives” that may make the interpretation difficult and flawed. In addition, redundancy in major biological pathways adds a further layer of complication in the interpretation of the bioinformatics of big data sets. These exercises may involve increased time management and, at times, may require multidisciplinary clinical/bioinformatics efforts to make a decisive treatment plan. According to a recent survey, the collection of huge data sets from patients potentially caused stress in clinicians, as it required a substantial amount of time for data interpretation and explanation to patients (231). In addition, patient information is sensitive and private and requires confidentiality and secured recording and storage. There are additional risks for patients to suffer from stigma and discrimination by insurance providers or employers if health records/data are made accessible or inappropriately disclosed. These can lead to serious ethical issues surrounding patients. For example, incidental detection of the presence of a life-threatening disease in an individual while genetic screening for one disease can have damaging impacts on the patient's physical and mental health, especially if there is no cure for the disease. To further complicate the issue, the rights of the patient's immediate family members or related members must also be considered in such situations. A genetic disease can be managed better with early intervention, and family members have the right to know if they are at any risk. However, it will mean encroaching patient's privacy and his/her right to disclose information to family members. Such an ethical dilemma is a huge concern for clinicians and patients practicing PM. In that context, the American Medical Association has formulated guidelines for physicians who require counseling patients about sharing the results of genetic tests with family members (232). The American Society of Human Genetics provides legislature to physicians to share the results of genetic tests that can pose health risks to family members if patients refuse to do so (233). However, this declared right given to treating physicians might result in unintentional aftereffects on families. For example, in cases of testing to determine potential birth effects in an unborn child, both parents undergo genetic testing. This, at times, can lead to paternity issues, which can have serious consequences for concerned families. All the above issues highlight the importance of legislative and confidentiality issues related to patients' health information and require due consideration while developing PM-oriented laws.

### Economic Impact of PM

The principal barrier to implementing PM in clinics is meeting the cost required for its execution. Economic evaluations of PM interventions for making policy decisions pertaining to investment in research and development (R and D) and reimbursement in healthcare systems have been suggested (234). PM has the potential to reduce the cost of healthcare as it can predict the right treatment for the right patient, thereby reducing any unnecessary and multiple trial-and-error attempts to achieve healthy patient outcomes. However, to reach this stage, huge investment in multiple directions is required, right from the secured and massive infrastructure to hold and collect patient data to the development of a precision drug. The R and D of PM can be costlier than traditional medicine because it requires the use of expensive techniques to test, like sequencing a large amount of DNA or RNA and other genetic testing. Hence, despite its potential to decrease the treatment time and unnecessary side effects due to the use of broad-spectrum drugs currently used in cancer therapy, the overall cost of healthcare may prove to be a burden for not-so-affluent patients and insurance payers. To put that in perspective, the US invested $ 215 million in funding toward PM out of $35 billion in health research and development in 2016 (235). The development of a drug is the costliest aspect of any healthcare system. It currently exceeds $2.7 billion for cancer drugs, which is regularly used as a justification for higher cancer drug prices (236). Reimbursement from health insurance companies will get tougher with the rise in patients' out-of-pocket costs. All these factors will have an indirect and a direct effect on patients and can mean higher costs for patients to avail PM. This will only lead to economic discrimination, with only the affluent sector of the public being able to afford the PM treatments. Strategic approaches need to be in place like changing regulations governing health insurance as well as pharmaceutical companies to overcome the cost challenges involved in bringing PM to deliver improved health outcomes to a broader sector of patients. A recent study showed that stratifying patients according to PM-based approaches could save approximately $7 million in overall healthcare costs per 1,000 patients (237). In that context, detection of HER2, a validated biomarker in a specific cohort of patients with breast cancer overexpressing HER2 in tumors, reduced the clinical trial risk by 50 and 27% reduction in cost (238). There are studies that convincingly prove that using precise-protocoled PM therapeutics with well-designed analytic strategies can reduce clinical trial risks in patients with cancer (239–241).

## Conclusions

The number of PM-based pharmaceutical drugs used in clinics has increased more than double from 2016 [132] to 2020 [286] (242). In that context, 31 genome-targeted anti-cancer drugs were used in 2018 [159]. The conventional cancer therapeutics reduce tumor burden and treas cancer-related symptoms, but relapse is common in most cancer cases. However, with the aid of genetic testing and the identification of specific protein biomarkers, more predictable patient health outcomes are possible.

**Figure 2:**
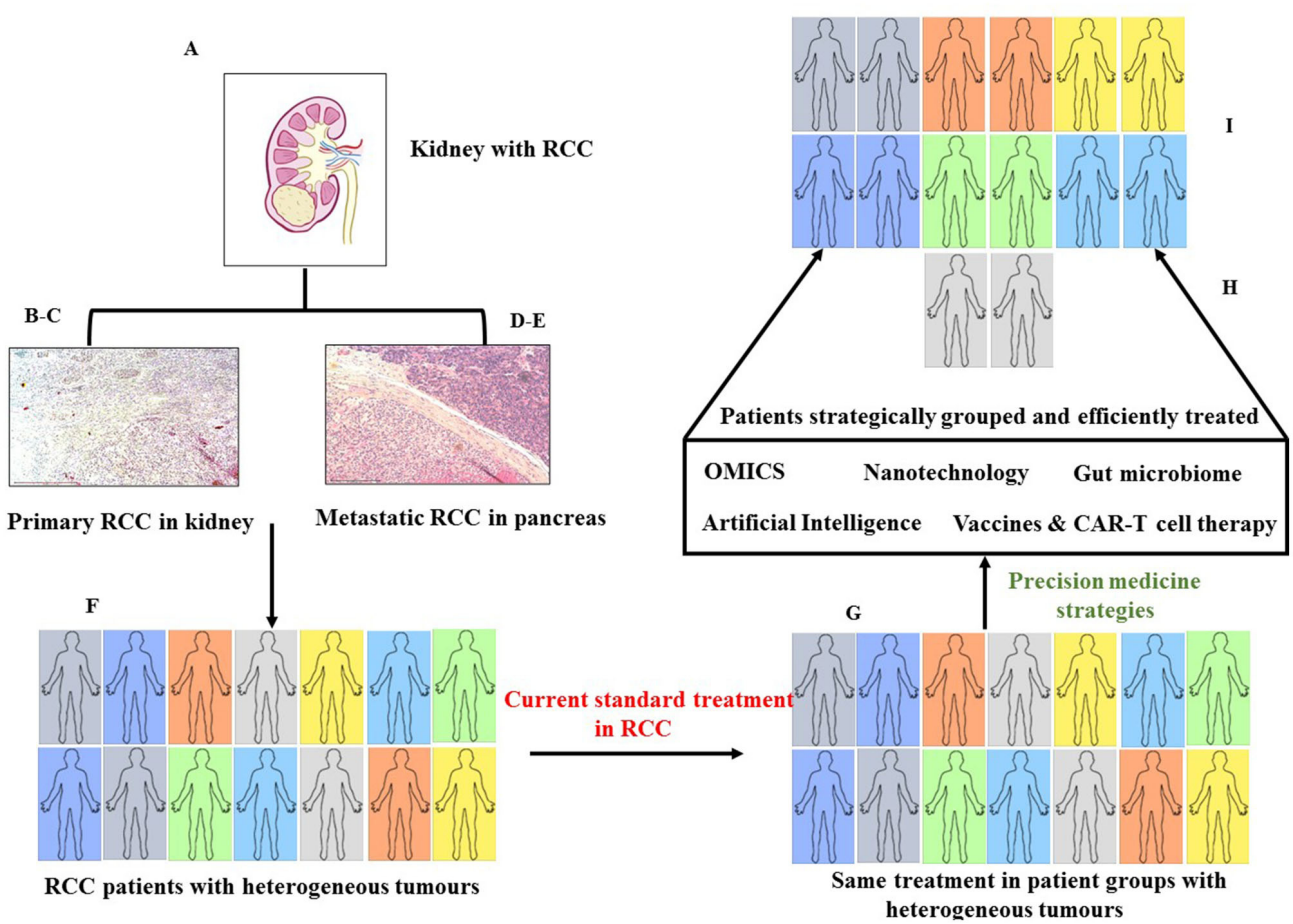
Role of precision medicine (PM) in RCC. **(A)** Hematoxylin and Eosin (H & E) staining of human kidney with RCC, **(B)** H & E of primary RCC showing typical epithelial nests of RCC cells with clear cytoplasm (Magnification: 100X), **(C)** adjacent normal kidney tissue, **(D)** metastatic RCC invading the pancreas of a patient tissue, **(E)** adjacent normal spleen in a patient tissue (Magnification: 100X). **(F)** heterogeneity in RCC patients due to diverse clinical and environmental factors, **(G)** current practice of treating patients – every patient treated with same standard drugs resulting in treatment failure and secondary resistance, **(H)** precision medicine approach utilizing data obtained from various platforms and stratifying patients with personalized treatments, **(I)** individualized treatment (PM) resulting in better clinical outcomes for patients.

RCC is a heterogeneous tumor at both the clinical and molecular levels. At the same time, RCC is a vascular, immunogenic, and metabolic tumor. The extensive variability in RCC tumor subtypes makes it a perfect candidate for PM, as the requirement for a patient-centered approach is high. Although much is known about RCC, the current treatment regimen relies highly on prognostic stratification of patients based on the assessment of clinical factors (243). Although several attempts were made to find accurate biomarkers to evaluate response to target therapies and immunotherapy, none of them have been successfully adopted for mass patients with RCC. Hence, finding validated genetic or molecular biomarkers to assist in clinical decision-making is of clinical priority and an important step in taking RCC toward the PM arena. The use of patient-derived organoids and PDX and the development of cancer vaccines and CAR-T cells are slowly but steadily changing the arena of current PM. This is pushing the boundaries of current treatments more toward individually focused treatment in RCC. With skyrocketing costs of healthcare, PM has a scope of providing personalized healthcare at affordable costs by cutting repeated trial-and-error treatment strategies, which not only take a heavy toll on patients' health but also affect the immediate families. While celebrating the accomplishments of PM is necessary, its failures should be addressed as well. Tight regulations should govern PM products and services to provide essential and cost-effective positive patient-oriented health outcomes. While conventional medicine is considered as a shotgun that shoots pellets in a wide range with the hope to hit the target in the line of fire, PM may mitigate its way as a laser with focus only on the precise targets. It has the potential to reduce the cancer-associated economic and social burden. While there is still a lot to be achieved in the struggle for PM in RCC treatment, which is far from reaching clinics, PM is the way that offers more specific and individualized treatments for patients. Figure 2 and Table 2 depicts the importance of PM in the treatment of RCC patients.

**Table 2:** Summary of applications of precision medicine (PM) to facilitate treatment of patients with renal cell cancer (RCC).

**Tools for personalized data collection**				
Lifestyle data	Genomics	Proteomics	Transcriptomics	Metabolomics
**New approaches to precision introduced in precision medicine**				
Artificial Intelligence		Gut Microbiology	Nanotechnology	
**Strategies of delivering PM in RCC**				
Vaccines		Cellular therapies & organoids	Monoclonal antibodies	

## Author Contributions

RS undertook the literature review, was involved with the conception of the idea, and wrote the article. PP and GK edited and read the final version of the manuscript. NA was involved in conceiving the idea and writing and editing the manuscript. All the authors have read and approved the manuscript.

## Funding

This study was supported by John Turner Cancer Research Funds to Fiona Elsey Cancer Research Institute, Ballarat, Australia. RS is a recipient of a John Turner Cancer Research PhD scholarship, Fiona Elsey Cancer Research Institute, and Federation University Australia, Ballarat, Australia. This study was made possible through funds from John Turner Cancer Research Funds and the Victorian State Government Operational Infrastructure Support to Hudson Institute of Medical Research.

## Conflict of Interest

The authors declare that the research was conducted in the absence of any commercial or financial relationships that could be construed as a potential conflict of interest.

## Publisher's Note

All claims expressed in this article are solely those of the authors and do not necessarily represent those of their affiliated organizations, or those of the publisher, the editors and the reviewers. Any product that may be evaluated in this article, or claim that may be made by its manufacturer, is not guaranteed or endorsed by the publisher.
